# Transcatheter Arterial Embolization of a Ruptured Bronchial Artery Aneurysm Presenting as Hematemesis: A Case Report

**DOI:** 10.2174/0115734056372341250628185446

**Published:** 2025-07-07

**Authors:** Gwanghyun Kim, Lyo Min Kwon, Young Soo Do, Kyung Sup Song, Wonju Hong

**Affiliations:** 1 Department of Radiology, Hallym University Sacred Heart Hospital, Anyang-si, Korea

**Keywords:** Hematemesis, ruptured aneurysm, bronchial arteries, therapeutic embolization, gastrointestinal hemorrhage, esophageal fistula

## Abstract

**Background:**

Hematemesis is a rare manifestation of a bronchial artery aneurysm (BAA), as bleeding from a ruptured BAA typically occurs into the bronchial tree, leading to hemoptysis rather than gastrointestinal bleeding.

**Case Presentations:**

A 71-year-old male presented to the emergency department with syncope and hematemesis. Computed tomography angiography (CTA) revealed a ruptured bronchial artery aneurysm in the posterior mediastinum, with contrast extravasation into the lower esophagus. The patient underwent transcatheter arterial embolization (TAE) using coils, a mixture of N-butyl cyanoacrylate and ethiodized oil. However, due to persistent bleeding signs and recanalization observed on follow-up CTA, a second TAE was performed the following day using the same technique. Hemostasis was achieved, and the patient recovered well, being discharged on the 16^th^ day without complications.

**Conclusion:**

Ruptured BAA presenting as hematemesis is extremely rare, making it difficult to diagnose. Prompt diagnosis with CTA and timely intervention, such as TAE, can be important in achieving favorable outcomes and preventing life-threatening complications.

## 1 INTRODUCTION

Bronchial artery aneurysm (BAA) refers to an aneurysmal dilatation of the bronchial artery. It is a rare condition, observed in less than 1% of all selective bronchial arteriographies [1]. Unruptured BAAs are asymptomatic and often discovered incidentally, or they may present with minor symptoms, such as chest discomfort or dyspnea [2]. When ruptured, BAAs usually lead to acute chest or back pain and hemorrhage, which manifest as hemomediastinum, hemothorax, or hemoptysis [1-3].

The etiology of BAAs still remains unclear. In one study, bronchiectasis was found to be the most frequently associated condition, followed by hypertension, chronic obstructive pulmonary disease, vasculitis, chronic pulmonary infections, tuberculosis, and trauma [4]. The pathogenesis of BAA may involve increased flow of bronchial arteries and compromised vessel wall integrity [1].

Since the risk of rupture does not appear to correlate with the size of the aneurysm, timely endovascular management is recommended regardless of symptom presence or aneurysm size [1-3, 5]. Hematemesis is an exceptionally rare presentation of BAA, with only a few cases reported in the literature [6, 7]. Here, we present a case of ruptured BAA presenting with hematemesis, successfully treated with two sessions of transcatheter arterial embolization (TAE).

## 2 CASE DESCRIPTION

**Fig. (1):**
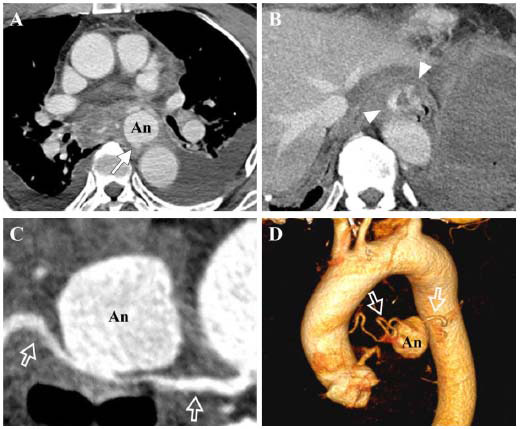
Contrast-enhanced computed tomography images in the venous phase of a 71-year-old man presenting with hematemesis. (**A**) A saccular aneurysm (An), approximately 3 cm in size, is seen in the posterior mediastinum with contrast extravasation through the posterior wall, suggesting a rupture (arrow). (**B**) Extravasated contrast medium is seen within the esophageal lumen (arrowheads). (**C**) Reconstructed image of the right bronchial artery (open arrows) using the centerline method shows connection with the aneurysm (An), indicating bronchial artery aneurysm. (**D**) Volume Rendering Technique (VRT) image clearly demonstrates the origin of the aneurysm (An) and the right bronchial artery (open arrows) from the thoracic aorta.

A 71-year-old male presented to the emergency department with approximately 300 mL of hematemesis and syncope. He had no known medical conditions or was not taking any medications. His initial blood pressure was 67/46 mmHg, and he appeared drowsy, with associated dizziness, chest discomfort, and epigastric pain. The initial hemoglobin level was 9.1 g/dL. An immediate blood transfusion was administered, and computed tomography angiography (CTA) was performed to identify the source of bleeding. CTA was performed using a dual-source CT scanner (SOMATOM Force, Siemens Healthineers, Germany) with a slice thickness and increment of 1.0 mm. A weight-based contrast agent protocol (1.8 mL/kg, flow rate 4.0 mL/s) was used with arterial and portal phases acquired at 8 and 30 seconds, respectively. Additional reconstructed images were generated using volume rendering techniques during the arterial phase, and radiation modulation tools, such as CARE Dose4D and CARE kV, were applied across all phases. CTA revealed a 3cm saccular aneurysm in the posterior mediastinum arising from the right bronchial artery, accompanied by a mediastinal hematoma, hemothorax, and contrast extravasation into the lower esophagus, findings consistent with a ruptured BAA and an esophageal fistula (Fig. **1**). Emergent esophagogastroduodeno-scopy revealed bluish discoloration of the lower esophageal mucosa and a mucosal tear at the gastric cardia, without evidence of active bleeding during the procedure. Due to the patient’s hemodynamic instability, emergent TAE of the BAA was performed.

**Fig. (2):**
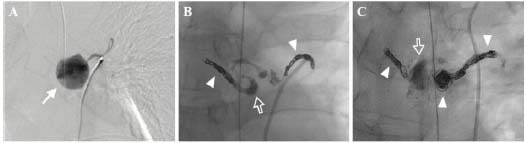
Fluoroscopy captured during two sessions of TAE for ruptured BAA. (**A**) A digital subtraction angiography showed a contrast-filled bronchial artery aneurysm (arrow). (**B**) The first session of TAE was done using coils (arrowheads) and glue (open arrow). (**C**) The aneurysmal sac was filled with additional coils (arrowheads) and glue (open arrow) after the second session of TAE. The patient showed no further bleeding or adverse events and recovered well after the procedure.

Through right femoral artery access, a 5-French catheter was inserted to approach the right bronchial artery. A 3cm saccular aneurysm was identified in the proximal portion of the right bronchial artery (Fig. **2A**). The distal segment of the aneurysm was successfully selected using a microcatheter, and multiple detachable coils (Concerto, Medtronic, Inc., Minneapolis, MN, USA) were deployed, with 30% oversizing relative to the diameter of the distal normal vessel. The aneurysmal sac was filled with a 1:5 mixture of N-butyl cyanoacrylate (NBCA; Histoacryl, B. Braun Medical Inc., Melsungen, Germany) and ethiodized oil (Lipiodol, Guerbet LLC, Princeton, NJ, USA), followed by coil embolization of the proximal portion using seven microcoils (Fig. **2B**). Post-procedural angiography confirmed successful embolization, with flow stasis at the proximal part of the aneurysm. The procedure took approximately one hour and was completed without any immediate complications.

However, after the procedure, the hemoglobin level did not increase despite transfusions, and bloody fluid continued to be aspirated *via* the nasogastric tube. A repeat CTA performed the day after the first TAE showed partial contrast filling, indicating recanalization of the aneurysm and right bronchial artery, along with subtle enlargement of the mediastinal hematoma, necessitating a second TAE. Angiography confirmed BAA recanalization. The second TAE was performed in the same manner as the first procedure. Eight additional detachable microcoils were deployed into the recanalized segment, followed by careful injection of a 1:5 mixture of NBCA and ethiodized oil under fluoroscopic guidance at a slow, controlled rate to ensure compact packing. Hemostasis was successfully achieved without complications (Fig. **2C**).

After the second TAE, the patient's hemoglobin levels gradually increased, and his symptoms improved. The mediastinal hematoma decreased in size, and the hemothorax resolved following percutaneous catheter drainage. Follow-up endoscopy on the 9^th^ and 15^th^ days after admission revealed healing ulcers without evidence of active bleeding. The patient was discharged on the 16^th^ day of hospitalization. Follow-up computed tomography (CT) scans performed at 1, 6, and 12 months demonstrated complete embolization, resolution of the hematoma, and no evidence of recanalization. At the 18-month outpatient follow-up, the patient remained asymptomatic with no signs of recurrence.

## 3 DISCUSSION

This report presents a very rare case of a ruptured mediastinal BAA causing life-threatening hematemesis due to an esophageal fistula. The bleeding was successfully controlled *via* an endovascular approach using coils and an NBCA mixture, and the patient recovered uneventfully. The patient had to undergo two sessions of TAE due to recanalization. This may have been associated with suboptimal packing using a Concerto coil, which has relatively fewer fibers and is less thrombogenic. In addition, the use of overly diluted glue may have resulted in reduced adhesive strength and a risk of migration, ultimately leading to incomplete occlusion of the flow. To prevent recurrence, it is essential to achieve compact embolization and ensure complete flow stasis on post-embolization angiography.

According to our literature review, BAA presenting with hematemesis is extremely rare, with only five cases reported in the English (Table **S1**). In all reported cases, the aneurysms were located in the mediastinum, and bleeding occurred in the mid to lower esophagus [5-9]. In four of the five cases, a fistula between the aneurysm and esophageal mucosa was identified [6-9], suggesting that hematemesis results from fistula formation between the aneurysm and the esophagus. In some reports, air density within the aneurysm on CT suggested communication with the gastrointestinal tract [7, 8]. Endoscopy may reveal the fistula directly or show extrinsic esophageal compression with active bleeding [6, 8]. Regarding treatment, three of the five cases were managed with TAE using coils and NBCA [5, 6, 8]. One patient required repeat embolization due to recanalization [8]. In another case, thoracic endovascular aortic repair (TEVAR), followed by minimally invasive surgical repair, was performed due to the presence of a short-neck aneurysm [9]. One patient died from a massive hemorrhage without undergoing any intervention [9].

BAAs are classified as either mediastinal or intrapulmonary based on anatomical location, which affects their clinical presentation [1, 2, 10]. Unruptured mediastinal BAAs could be asymptomatic or cause compressive symptoms, such as dysphagia or superior vena cava syndrome [1, 3, 11], while ruptures can result in hemomediastinum or hemothorax [1-3, 9, 12]. In rare instances, they may present with hematemesis, as demonstrated in our case. The vascular pressure from BAAs may cause erosion in the esophagus due to their anatomical proximity and sustained pressure without underlying esophageal pathology itself [13]. Hematemesis from a ruptured BAA could be more massive and potentially fatal due to the high-pressure arterial flow. On the other hand, unruptured intrapulmonary BAAs are often asymptomatic, but when they rupture, they typically present with hemoptysis, ranging from mild to massive [1, 3].

Timely diagnosis of ruptured BAA is crucial due to the risk of fatal hemorrhage [2]. Endoscopy remains the first-line approach in most cases of hematemesis. However, in hemodynamically unstable patients like our case or when endoscopy is inconclusive, early CTA should be considered to evaluate for rare but critical vascular causes, such as aneurysms or fistulas. On CTA, BAAs usually appear as saccular contrast-enhancing lesions connected to either the right or left bronchial artery and may contain thrombi [2, 3, 5-7]. The aneurysm size varies widely from 3 mm to 70 mm, and multiple lesions may occur in a single patient [3, 6].

Treatment is recommended upon diagnosis, regardless of symptoms or aneurysm size [1]. TAE is regarded as the first-line treatment because it is minimally invasive and can be performed without general anesthesia [5, 14]. Embolization is usually achieved with coils, and NBCA can be used as an adjunct agent [1]. However, TAE may not be feasible in cases where a microcatheter cannot be adequately advanced to both the proximal and distal segments of the aneurysm, or when the proximal portion of the feeding artery is too short for stable embolization. In such situations, alternative approaches, such as TEVAR or surgery, could be considered [15].

## CONCLUSION

In conclusion, we report a rare case of ruptured mediastinal BAA causing hematemesis secondary to an esophageal fistula, successfully treated with TAE. Massive hematemesis may, in rare cases, originate from arterial sources, such as BAA, and prompt CTA can be crucial for timely diagnosis. For effective embolization, compact packing and confirmation of complete occlusion are essential to minimize the risk of recanalization.

## Data Availability

All data generated or analyzed during this study are included in this published article.

## LIST OF ABBREVIATIONS

BAA: Bronchial Artery Aneurysm
CTA: Computed Tomography Angiography
CT: Computed Tomography
TAE: Transcatheter Arterial Embolization
NBCA: N-butyl Cyanoacrylate
TEVAR: Thoracic Endovascular Aortic Repair
VRT: Volume Rendering Technique

## STANDARDS OF REPORTING

CARE guidelines were followed.
