# Supplementary material for: Transcatheter Arterial Embolization of a Ruptured Bronchial Artery Aneurysm Presenting as Hematemesis: A Case Report
Source: Curr Med Imaging. 2025 Jul 7;21:e15734056372341. doi: 10.2174/0115734056372341250628185446 (PMC13223448; doi:10.2174/0115734056372341250628185446)
Supplement: Supplementary file 1 [file CMIM-21-E15734056372341_SD1.pdf]

1573-4056/25

Send Orders for Reprints to reprints@benthamscience.net

BENTHAM SCIENCE

CrossMark

Current Medical Imaging

Content list available at: https://benthamscience.com/journals/cmir

Current Medical Imaging

Transcatheter Arterial Embolization of a Ruptured Bronchial Artery Aneurysm Presenting as Hematemesis: A Case Report

Gwanghyun Kim[1], Lyo Min Kwon[1,*], Young Soo, Do[1], Kyung Sup Song[1] and Wonju Hong[1]

[1]*Department of Radiology, Hallym University Sacred Heart Hospital, Anyang, Korea*

Table S1. Reported bronchial artery aneurysms presenting with hematemesis.

Case	Age/Sex	Associated Symptoms other than Hematemesis	Location/Size	Radiologic Findings	Endoscopic Findings	Treatment (Used Materials)	Prognosis
Shaer *et al.* [9]	79/M	None*	Mediastinum, right bronchial artery / 10cm on autopsy	Non-CE CT: 7cm soft tissue mass abutting the descending aorta	Not performed	None	Expired
Fukunaga *et al.* [8]	60/M	Anterior chest pain, back pain, dysphagia	Mediastinum, right bronchial artery / 4cm	CE CT: Low density mass with partial enhancement, air inside the aneurysm on F/U study<br>MRI: heterogeneous intensity on T1 and T2, signal void corresponding to enhancing lesion on CT	Post-treatment: swelling lesion in esophagus with a clot-discharging pinhole in the middle	TAE (Coils)	No relapse after 4 years
Kim *et al.* [5]	55/M	Acute chest pain, neck swelling, dysphagia, dyspnea	Mediastinum, right bronchial artery / 1cm	Chest X-ray: right superior mediastinal widening<br>CE CT: mediastinal hematoma, aneurysm visible on F/U study	Extrinsic esophageal compression	TAE (Coils, NBCA)	No recurrence after 1 year
Nakada *et al.* [7]	67/M	None†	Mediastinum / 5cm	CE CT: saccular aneurysm adjacent to the descending thoracic aorta, air inside the aneurysm on F/U study	Extrinsic mass of the esophagus with oozing blood<br>Post-treatment: mucosal degeneration with a fistula	TEVAR followed by minimally invasive surgery	Well after over 1 year
Alharbi SR. [6]	19/M	Epigastric pain, chronic cough, weight loss	Bronchial artery arising from left subclavian artery / Not mentioned	CE CT: 2 small mediastinal bronchial artery aneurysms with esophagomediastinal fistula‡	Opening in the mid-esophagus without clot	TAE (Coils), anti-TB medications	Asymptomatic after 3 months

Notes*: hematemesis occurred 3 weeks after ingesting household ammonia; †: hematemesis occurred 3 months after previous BAE for hemoptysis; ‡: with multiple necrotic mediastinal lymph nodes and cavitary lung lesion, which revealed as manifestation of mediastinal tuberculosis; CE CT: contrast-enhanced computed tomography; F/U: follow-up; TAE: transcatheter arterial embolization; NBCA: N-butyl cyanoacrylate; TEVAR: thoracic endovascular aortic repair; TB: tuberculosis.

© 2025 The Author(s). Published by Bentham Science Publisher.

This is an open access article distributed under the terms of the Creative Commons Attribution 4.0 International Public License (CC-BY 4.0), a copy of which is available at: https://creativecommons.org/licenses/by/4.0/legalcode. This license permits unrestricted use, distribution, and reproduction in any medium, provided the original author and source are credited.

DOI: 10.2174/0115734056372341250628185446, 2025, *21*, e15734056372341
